# An alternative to mineral phosphorus fertilizers: The combined effects of *Trichoderma harzianum* and compost on *Zea mays*, as revealed by ^1^H NMR and GC-MS metabolomics

**DOI:** 10.1371/journal.pone.0209664

**Published:** 2018-12-27

**Authors:** Giovanni Vinci, Vincenza Cozzolino, Pierluigi Mazzei, Hiarhi Monda, Riccardo Spaccini, Alessandro Piccolo

**Affiliations:** Centro Interdipartimentale di Ricerca sulla Risonanza Magnetica Nucleare per l’Ambiente, l’Agroalimentare ed i Nuovi Materiali (CERMANU), Università di Napoli Federico II, Portici (NA), Italy; Universita degli Studi di Pisa, ITALY

## Abstract

The ability of *Trichoderma harzianum* (strain OMG-08) as plant growth promoting fungus (PGPF), was tested on *Zea mays* plants grown in soil pots added with different inorganic (triple superphosphate and rock phosphate) and organic (cow and horse manure composts) P fertilizers. The effect of treatments was evaluated by following the variations of plants dry biomass and nutrient content, as well as the metabolic changes in plant leaves by both GC-MS and NMR spectroscopy. A synergic effect was observed in treatments with both composts and fungus inoculation, in which not only plant growth and P uptake were enhanced, but also the expression of different metabolites related to an improved photosynthetic activity. Conversely, the combination of *Trichoderma* with inorganic fertilizers was less effective and even showed a reduction of plants shoot biomass and N content. The corresponding plant metabolome revealed metabolic compounds typical of biotic or abiotic stresses, which may be attributed to a reduced capacity of inorganic fertilizers to provide a sufficient P availability during plant growth. Our findings also indicate that the molecular composition of compost differentiated the *Trichoderma* activity in sustaining plant growth. The positive effects of the combined *Trichoderma* and compost treatment suggest that it may become an alternative to the phosphorus mineral fertilization.

## Introduction

Conventional farming and its basic common practices still guarantees a sufficient crop production to match the present demand for human food. However, total population will reach around 10 billion people in the next 50 years, thus making mandatory a further increase in food production [1]. While the conventional farming will be called to keep pace with worldwide population growth, the implied intensive applications of N and P mineral fertilizers and chemical pesticides may negatively influence the structure and composition of soil micro-flora [2, 3, 4], thereby leading to a progressive reduction of soil fertility and crop yields [5]. Moreover, the reserves of rock phosphate (RP), which provide the raw material to produce inorganic fertilizers, including the Triple Superphosphate (TSP), are forecasted to be drastically depleted by the end of this century [6]. The inevitable raise on the cost of these fertilizers calls for the development of innovative low-cost and eco-sustainable crop nutrition practices [7].

Organic farming is the alternative that, by integrating the use of recycled organic matter with microbial bio-stimulants, not only ensures food safety but also adds biodiversity to soil [8, 9]. Compost represents the most utilized form of stabilized recycled biomass as an efficient soil amendment [10, 11]. Furthermore, compost made of animal manure is an inexpensive source of bioavailable P for plants and it is progressively used in substitution of or in combination with inorganic fertilizers [4, 11].

The capacity of soil microorganisms to affect plant growth depends on sophisticated nutritional and chemical signaling, but also on soil conditions and climate factors [12]. Among microbial biostimulants, plant growth promoting fungi (PGPF) received considerable attention within the development of a sustainable agriculture. In particular, *Trichoderma* species are known to establish beneficial interactions with plants, since its symbiosis with crops may promote crop yields [13, 14]. *Trichoderma spp*. may also act as biocontrol agents [13], as well as inducers of disease resistance in symbiotic plants [15, 16]. These beneficial properties are accomplished via modulation of root architecture or through exudation of substances capable to increase plant access to scarce or not available nutrients in soil, including phosphorus [17, 18]. The positive effects of the *Trichoderma*-plant interactions are expected to be reflected in substantial changes of metabolites concentration in plants, thus affecting the metabolome [16, 19], proteome and transcriptome [20, 21, 22] of the symbiotic plant.

Therefore, a metabolomic approach was used here to evaluate the induced metabolic changes on *Zea mays* plants treated with *Trichoderma* fungi either alone or in combination with inorganic (TSP, RP) and organic (two composts) fertilizers containing the same amount of available P. In particular, we applied both gas chromatography coupled to mass spectrometry (GC-MS) and Nuclear Magnetic Resonance (NMR) spectroscopy to identify the polar metabolites extracted from plant leaves and correlate the results with changes in plant growth, chlorophyll, and P and N content. Furthermore, a detailed characterization of individual molecular components of both composts was performed by off-line thermochemolysis coupled with GC-MS. The qualitative-quantitative content of specific classes of compounds were related to the *Trichoderma* activity and different effects on plants.

## Materials and methods

### Materials

A surface layer (0 to 20 cm) of a Vertic Xerofluvent clay-loam soil was sampled at the experimental station of the Università di Napoli Federico II located in Castel Volturno (CE), Italy. S1 Table reports the principal physical-chemical characteristics of soil. The inorganic fertilizers, TripleSuperPhosphate (TSP) (P1) and Rock Phopshate RP (P2), were provided by Landor AG, Birsfelden, Switzerland, and Herbert Molitor, Germany, respectively. The cow-(P3) and horse-(P4) manure composts were produced at the composting plant of the experimental station of Castel Volturno. The elemental composition showed a content of 30% C, 2.3% N, and 7.75 g kg^-1^ P in P3, while P4 contained 31% C, 1.4% N, and 10.0 g kg^-1^ P. The fungus bioeffector (B2), *Trichoderma harzianum* (OmG-08) was provided, within the EU-BIOFECTOR project (7^th^ FP) by the Anhalt University of Applied Science, Germany. Additional information’s on BIOFECTOR project and the bioeffectors used therein are available on the web site: http://www.biofector.info.

### Pot experiment: Experimental design, plant growth, sampling and analyses

The pot experiment of this study was conducted as previously reported [23, 24]. Briefly, the maize plants (*Zea mays* cv. *Colisee*, KWS) were grown in pots (3 L) using 2.5 kg of mixed substrate (soil and quartz sand in the 2:1 ratio). Different P fertilizers were used to enrich in a factorial combination the substrate: a) P0 no addition; b) P1, TSP; c) P2, RP; d) P3, compost from cow manure; e) P4, compost from horse manure. All fertilizers were added to guarantee a final concentration of 50 mg P kg^-1^ dry soil substrate mixture in the P1-P4 pots. The B0 code indicates the absence of microbial bioeffectors, while the B2 code indicates treatment with *Trichoderma harzianum* (OmG08). Five replicates for each treatment were prepared. Additionally, Ca(NO_3_)_2_, and Kalimagnesia (30% K_2_O + 10% MgO) were added as basal nutrients, nitrogen (N) and potassium (K), to obtain a final concentration of 150 mg N and 166 mg K for kg^-1^ of dry soil substrate mixture in the P0-P4 pots.

*Trichoderma* was applied as a spore suspension in deionized water with 2.5 mM CaSO_4_, and was sprayed on the seeds surface at sowing, at the rate of 1.5 x 10^5^ spores g^-1^ dry substrate. The water holding capacity of substrate mixture was maintained between 40 and 70% throughout the experiment. Sampling was performed eight weeks after sowing, collecting the youngest and fully expanded leaves. The leaf samples were immediately weighed and frozen in liquid nitrogen prior to be stored at -80°C. The P content in maize shoots was measured as by the method reported elsewhere [25, 26], while the nitrogen concentration was determined by the elemental analyzer Fisons EA 1108 (Fisons Instruments, Milano, Italy). The chlorophyll content was calculated according to Lichtenthaler [27]. The two-way ANOVA followed by Tukey’s test, at a significance level of 5%, was performed to analyze the plant shoots biomass, and the chlorophyll and P, N contents in leaves.

### Extraction of metabolites from plant leaves

The polar metabolites were extracted from 50±0.5 mg of pre-homogenized leaves under liquid nitrogen, using 1ml of cold solvents mixture (water, methanol and chloroform in a ratio of 1:3:1). The ribitol was used as internal standard in a concentration of 20 μg mL^-1^. The samples were stirred for 30 s and incubated for 15 min at 70°C in order to inhibit the activity of possible enzymes present in the extract, and, then, centrifuged for 10 min at 10000 rpm at 4°C to separate the supernatants from pellets. Milli-Q water (400 μL) was added to supernatants in order to separate the polar and apolar phases. Finally, 400 and 800 μL from the polar phase were transferred into 2 different GC–MS glass vials, dried under a nitrogen flow and stored at -80°C before the analysis.

### Analytical determination of metabolites

Before derivatization for GC–MS analyses, the vials containing 400 μL of dried extracts were dissolved in 50 μL of a pyridine solution containing 20 mg mL^-1^ of methoxyamine hydrochloride. The reaction of methoximation was obtained by treating the samples for 90 min at 30°C. After this step, samples were silylated for 30 min at 37°C by using 50 μL of N-methyl-N-trimethylsilyltrifluoroacetamide (MSTFA) reagent. 2 μL of the silylated solution were finally injected into a GC column.

In the case of NMR analysis, 800 μL of dry polar extracts were resolubilised in the same volume of deuterated phosphate buffer (90 mM, pH 6.0) containing 0.05 mg mL^-1^ of 3-(tri-methylsilyl) propionic-2,2,3,3-d4 acid (TMSPA, δ^1^H = 0 ppm; > 99%, Euriso-Top, France) as internal standard. Five replicates were prepared and examined by both NMR and GC-MS methods.

### GC-MS

Samples were analyzed by quadrupole type GC–MS system composed by an Agilent 7683B Series Injector coupled to an Agilent HP6890 Series gas chromatograph system and a 5973 Mass Selective Detector. The GC separation was performed with RTX-5MS WCOT capillary column (Restek, 30 m × 0.25 mm; film thickness, 0.25 mm), by applying a 2 minutes long isothermal phase at 80°C, followed by a temperature increase from 80 to 310°C (rate of 15°C min^-1^) and culminating in a 10 minutes long isothermal phase at 310°C. The helium gas was set at 1 mL min^−1^, while the injector temperature at 250°C and the split flow was 25 mL min^−1^. Mass spectra were obtained in EI mode by scanning in the range from 50 to 650 m/z, with a cycle time of 0.2 scan s^−1^. The compounds identification was carried out by the mass comparison with real standard or with the mass spectra libraries of both NIST 05 (http://www.nist.gov) and Max-Planck-Institute for Plant Physiology of Golm (Germany, http://csbdb.mpimp-golm.mpg.de/csbdb/dbma/msri.html.

### NMR

The NMR analyses were performed by a 400 MHz Bruker Avance spectrometer equipped with a 5 mm BBI Bruker probe and working at the ^1^H frequency of 400.13 MHz. Monodimensional ^1^H spectra were acquired by setting 5 s of thermal equilibrium delay, a 90° pulse length ranging within 8 and 8.85 μs (−2 dB of attenuation), 128 transients, 32768 time domain points, and 16 ppm (6410.3 Hz). The signal of residual water was suppressed by applying the on-resonance pre-saturation during thermal equilibrium delay.

The NMR signals were assigned through 2D NMR spectra of both samples and real standard compounds. The interpretations were confirmed also using the specific literature. 2D NMR spectra consisted of ^1^H–^1^H homo-nuclear experiments, such as COSY (Correlation SpectroscopY), TOCSY (Total CorrelationSpectroscopY) and NOESY (Nuclear OverHauser SpectroscopY), and hetero-nuclear ^1^H–^13^C experiments, such as HSQC (Hetero-nuclear Single-Quantum Correlation) and HMBC (Hetero-nuclear Multiple Bond Correlation). 2D experiments were acquired with spectral widths of 16 (6410.3 Hz) and 300 (30186.8 Hz) ppm for ^1^H and ^13^C nuclei, respectively, and a time domain of 2048 points (F2) and 256 experiments (F1). The homo-nuclear 2D spectra consisted in 16 dummy scans and 64 total transients. Additionally, a mixing time of 80 ms and a trim pulse length of 2500 ms were set for TOCSY experiment. HSQC and HMBC hetero-nuclear experiments were acquired with 16 dummy scans, 80 total transients and 0.5 μs of trim pulse length. These experiments were optimized by assuming 145 and 6.5 Hz as the optimal ^1^H –^13^C short and long range J-couplings, respectively.

Bruker Topspin Software (v 2.1, BrukerBiospin, heinstetten, Germany) and MNOVA Software (v.9.0, Mestrelab Research, Santiago de Compostela, Spain) were used to process the spectra. The free induction decays (FIDs) of 1D ^1^H spectra were Fourier transformed with a function size of 32768 points and applying a 0.3 Hz apodization. Both mono- and bi-dimensional spectra were phase- and baseline corrected.

### Statistical analysis of GC-MS and NMR results

The semiquantitative evaluation of metabolites was performed by normalizing each GC-MS peak area to the area of the internal standard (ribitol) and further modulating it as a function of sample fresh weight. ^1^H NMR spectra were divided in symmetrical *n*-intervals of 0.04 ppm, which were integrated and normalized to the internal standard (TMSPA).

The Principal Component Analysis (PCA) was used here to reduce the dimensionality of the multivariate dataset and concomitantly preserve the useful information expressed in terms of variable variance. The XLStat software, version 9.0 (Addinsoft) was used to process the PCA of the total dataset composed of 49 and 244 variables deriving from both GC-MS and NMR spectra, respectively. Significant differences in metabolites amounts as a function of the applied treatments were tested by one and two-way ANOVA, followed by Tukey’s test (significant for *p-*values < 0.05 at a significance α of 0.05). The loading vectors involved in different treatments were reported along the borders of each score-plot with name and orthogonal direction of most significant (ANOVA Tukey’s test) metabolites.

Heatmaps were developed through the Heatmapper software [28] by elaborating the most discriminating (ANOVA Tukey’s test) variables. Each score in the heatmap visualization represents the average value of 5 replicates. In the case of metabolites developing multiple NMR signals, elaboration was conducted by considering the average intensity of compound peaks (possibly excluding overlapped signals) and accounting for both multiplicity and protons quantity. In order to enhance the heatmap color modulation, the values were centered, for each comparison, to the value of the respective control and scaled, for each variable, to the specific range of variation for each variable.

### Thermochemolysis-GC-MS of compost

The off-line-thermochemolysis-GC-MS was performed as reported by Spaccini et al. [29]. Briefly, 0.5 g of compost was treated with 1 mL of tetramethylammonium hydroxide (TMAH) (25% in methanol) solution. After two hours the dried mixture was placed in a Pyrex tubular reactor and heated at 400°C for 30 min in a circular oven (Barnstead Thermolyne 21100 Furnace, Barnstead International, Dubuque, IA, USA). The gaseous products of thermochemolysis were flowed into two chloroform traps in series, which were kept in ice/salt baths. The resulting chloroform solutions were combined and dried. The residue was dissolved in 1 mL of chloroform and directly injected to GC-MS. The GC-MS analyses were conducted with a Perkin Elmer Autosystem XL by using a RTX-5MS WCOT capillary column (Restek, 30 m × 0.25 mm; film thickness, 0.25 mm) that was coupled, through a heated transfer line (250°C), to a PE Turbomass-Gold quadrupole mass spectrometer. The chromatographic separation was achieved with the following temperature program: 60°C (1 min. isothermal), rate 7°C min^−1^ to 320°C (10 min. isothermal). Helium was used as carrier gas at 1.6 mL min^−1^, the injector temperature was at 250°C, and the split-injection mode had a 30 mL min^−1^ of split flow. Mass spectra were obtained in EI mode scanning in the range 45–650 m/z, with a cycle time of 0.2 s. Compounds identification was based on comparison of mass spectra with the NIST-library database, published spectra, and real standards.

## Results and discussion

### Impact of P-fertilizers and *Trichoderma* on shoots dry weight and nutrient uptake

**Fig 1:**
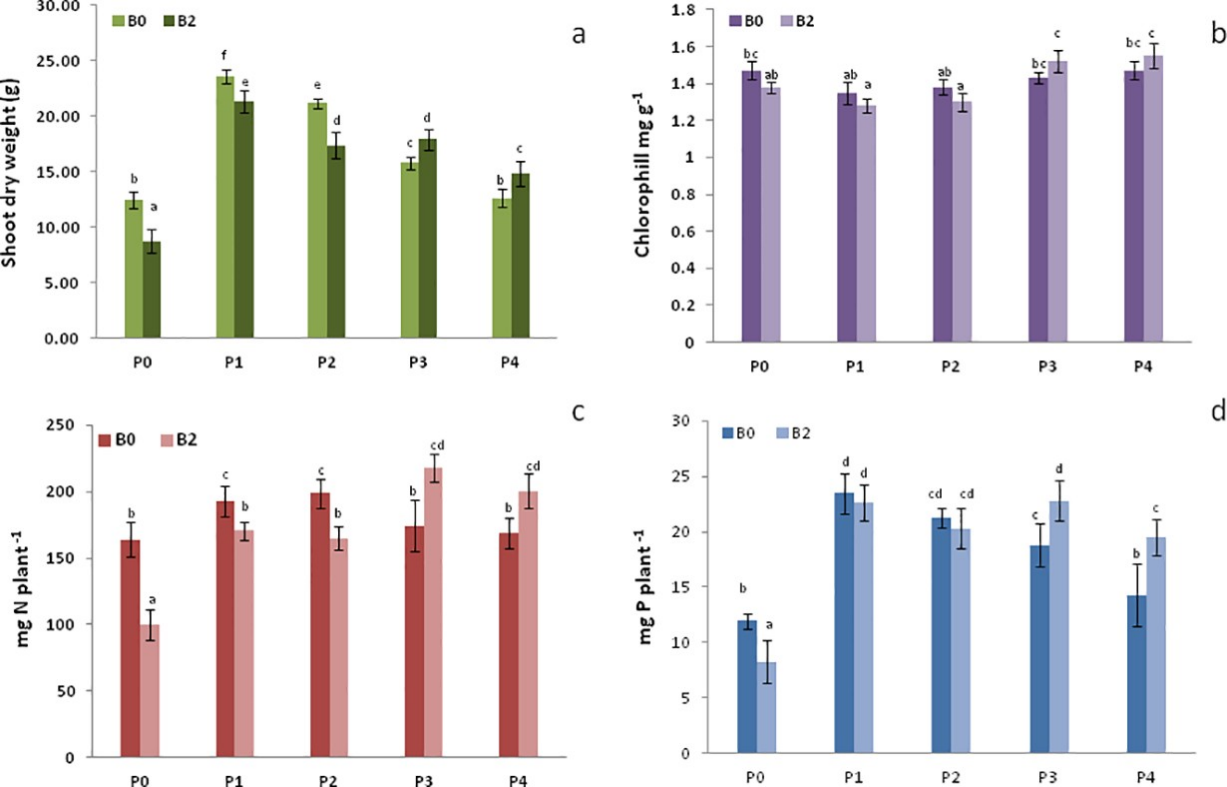
Plant shoots dry weight, chlorophyll, P and N content. Plant shoots dry weight (a), chlorophyll content (b), leaf phosphorus content (c), leaf nitrogen content (d), in control (B0) and inoculated maize plants (B2) treated with different P-based fertilizers (P0-P4). Error bars indicate standard deviation (n = 5) and different letters indicate significant differences by the Tukey’s test (*p* ≤ 0.05 at a significance of 0.05).

The impact of *Trichoderma* inoculum and different P fertilizations on plant growth and nutrition was evaluated by measuring dry biomass and P and N content in maize shoots at eight weeks after sowing. These parameters were positively influenced by P fertilizations alone or in combination with *Trichoderma*, in respect to the P0 control (Fig 1). In particular, the largest effect on plants was obtained with the TSP treatment (B0P1) that showed the greatest shoot dry biomass.

The addition to plants of *Trichoderma* alone without P fertilization (B2P0) reduced the leaves content of P and N, and the shoots dry weight (Fig 1A, 1C and 1D). The available soil P content measured by the Olsen method in the mixed substrate resulted to be 12 mg kg^-1^. This represents a low-moderate value for maize growth in the soil used for the experiment, in which more than 60% of soil P_i_ is bound to Ca compounds [24]. Li et al. [17] reported that during P-deficient hydroponic conditions, the *Trichoderma harzianum* (strain SQR-T037) adversely affected the tomato plant growth with a 82% biomass reduction. The authors explained the phenomenon with the competition between *Trichoderma* and plants for the scarcely available nutrients. In particular, the competition for P sources (e.g.: phytate) may induce the *Trichoderma harzianum* strain to suppress the development of plant roots. De Santiago et al. [30] also reported that *Trichoderma* (strain T34) significantly reduced the content of microelements (Cu, Zn and Mn) in the aerial parts of wheat grown under conditions of nutrients deficiency. Similarly, it can be hypothesized also in this study that the B2P0 treatment may cause a competition for the nutrient between *Trichoderma* (strain 0mG-08) and maize plants.

Conversely, variable effects were shown when plants were inoculated in combination with inorganic or organic P-fertilizers. The combination of *Trichoderma* with both inorganic fertilizers (B2P1, B2P2) significantly reduced the plant shoots biomass and the N content in comparison with the respective controls B0P1 and B0P2 (Fig 1A and 1C). These negative effects on plant should be related to the specific characteristics of different P fertilizers. In fact, P released in the rhizosphere by TSP and RF is quickly converted into insoluble forms [31], causing a strong competition for its solubilization between microorganisms and plants. Conversely, the combination of *Trichoderma* and composts (B2P3, B2P4) enhanced the shoot dry weight and P uptake to a larger extent than the corresponding B0P3 and B0P4 treatments (Fig 1A and 1B). In particular, the B2P3 treatment conferred to plants the best nutritional status in terms of P, N and chlorophyll content as compared to all other treatments (Fig 1).

These findings indicate that the positive synergistic action played by the combined *Trichoderma*/compost treatments, was probably due to the compost role as a substrate for microbial growth, thus limiting competition with plants. Therefore, the *Trichoderma* strain could exert its growth promoting action both directly by stimulating plant root development, and, indirectly, by contributing to solubilized mineral nutrients [30, 32].

### Effects of different treatments on maize leaves metabolome

Both GC-MS and ^1^H NMR spectroscopy were applied to identify the metabolites extracted from maize leaves grown under treatments with different P fertilizers alone or in combination with a *Trichoderma* inoculum. The main identified compounds were saccharides (mono- and di- saccharides), organic and amino acids (S2 and S3 Tables). In order to detect the effects of treatments on leaves metabolomes, the information obtained by both GC-MS and NMR analyses of plant leaves extracts were merged into a single matrix and semi-quantitatively evaluated by Principal Components Analysis (PCA).

**Fig 2:**
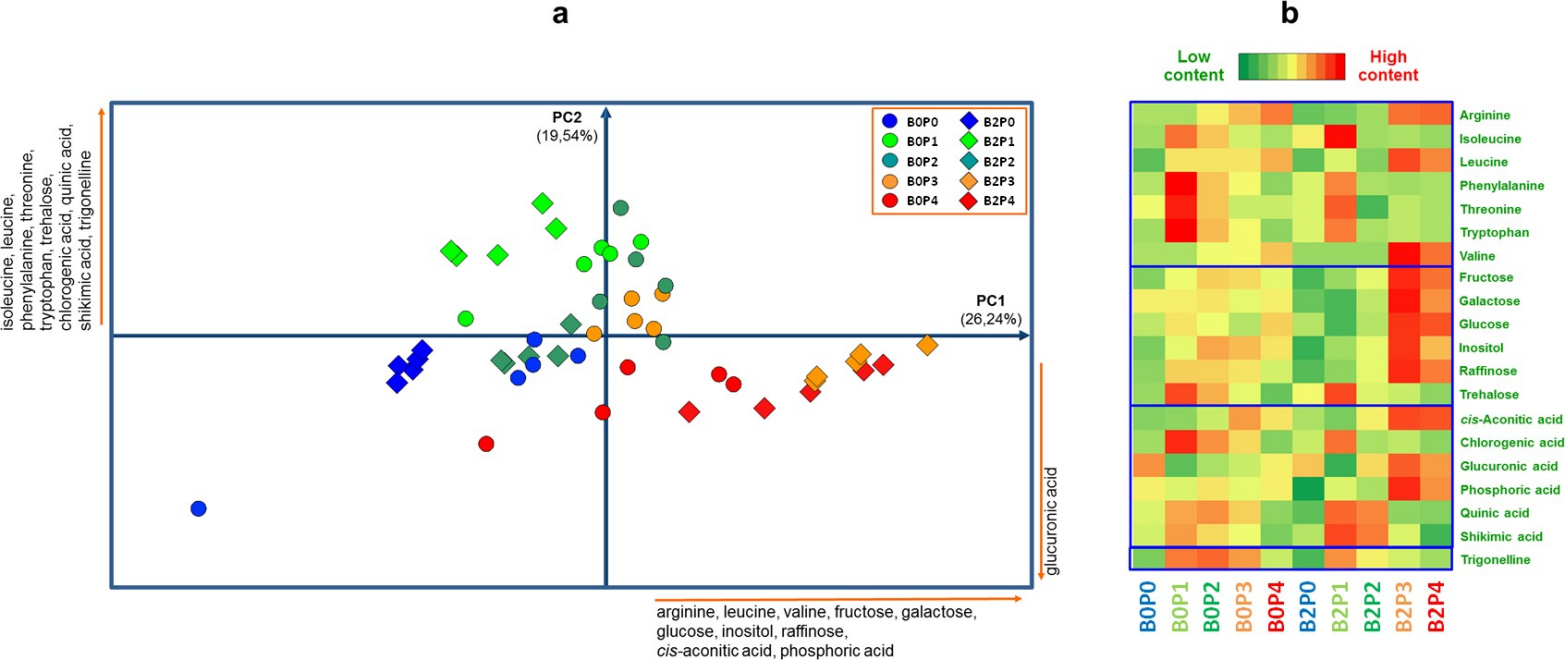
PCA and Heatmap of plant metabolites as a function of different treatments. (a) PCA score-plot based on GC-MS and ^1^H NMR data of leaves harvested from maize plants treated with different P-based fertilizers (P0-P4), with and without microbial inoculum (B2, B0). Names and direction of most significant PCA loading vectors involved in the differentiation of treatments are reported along the score-plot borders. (b) Heatmap visualization of most discriminant variables.

The PCA score-plot of metabolomes produced by the treatments accounted for 45.78% of the total variance and did show a degree of separation among treatments (Fig 2A). In particular, PC1 allowed to distinguish the B2P3 and B2P4 treatments from other ones. This differentiation was statistically significant and was due to more abundant metabolites such as glucose, fructose, galactose, phosphoric acid, raffinose, inositol, cis-aconitic acid and aminoacids arginine, leucine and valine. Similar results were found by also applying the Heatmap elaboration of the main metabolites (Fig 2B).

The largest metabolite concentration of sugars (glucose and fructose) and amino acids, as final products of photosynthesis and protein synthesis, respectively, well correlated with the large content of P, N and chlorophyll in the maize leaves of B2P3 and B2P4 treatments (Fig 1B–1D). Interestingly, the abundance of phosphoric acid (not a plant metabolite *sensu stricto*) appears to confirm the improved P uptake by plants and consequently an enhanced photosynthesis in treatments with both compost and *Trichoderma*. The presence of phosphoric acid should be accounted to a residual phosphatase activity in plant extracts that enabled the hydrolysis of both glucose- and fructose-6-phosphate into dephosphorilated sugar and inorganic P (detected by GC-MS as phosphoric acid) [33]. Among the aminoacids which were found to discriminate B2P3 and B2P4 from other treatments, arginine well correlated with the increased N content in plants since it represents an efficient form of organic N storage, due to the greatest nitrogen to carbon ratio of all others aminoacids [34].

The placement of B2P0 along the negative values of PC1 (Fig 2A) was due to a lesser amount of the main identified variables in the corresponding plant extracts, such as sugars (glucose and fructose), and amino acids (arginine, leucine, isoleucine, and valine), as also indicated by the derived Heatmap (Fig 2B). These results are in line with the smaller content of P, N and chlorophyll found in B2P0 plants (Fig 1) and suggested a less efficient photosynthesis and a reduced C and N metabolism, which both negatively affected plant growth [35, 36, 37, 38]. The B0P3 treatment and, to a lesser extent, the B0P2 and B0P4 ones showed intermediate values of all expressed metabolites along the PC1 component, and in the corresponding Heatmap (Fig 2A and 2B). These observations were in line with the intermediate values of plant biomass produced by these treatments and their relative nutritional content (Fig 1).

The increasing content along the positive values of PC2 of compounds such as phenylalanine, chlorogenic acid, quinic acid and trigonelline for the metabolomes of B2P1-treated plants, and, to a lesser extent, of B0P1 and B0P2, may be due to stress conditions induced in plants treated with inorganic fertilizers [39]. As reported by Vogt [40], large amount of phenylalanine can be produced during the synthesis of secondary metabolites such as lignin monomers, phenols and anthocyanins by the phenylpropanoid pathway. Therefore, the increased content of shikimic, quinic and chlorogenic acid confirms the activation of the lignin pathway [41]. The accumulation of these metabolites inside the vacuoles or in the apoplast occurs generally during leaf aging or under stress conditions [42].

### Effects of *Trichoderma* inoculation on maize leaves metabolomes

**Fig 3:**
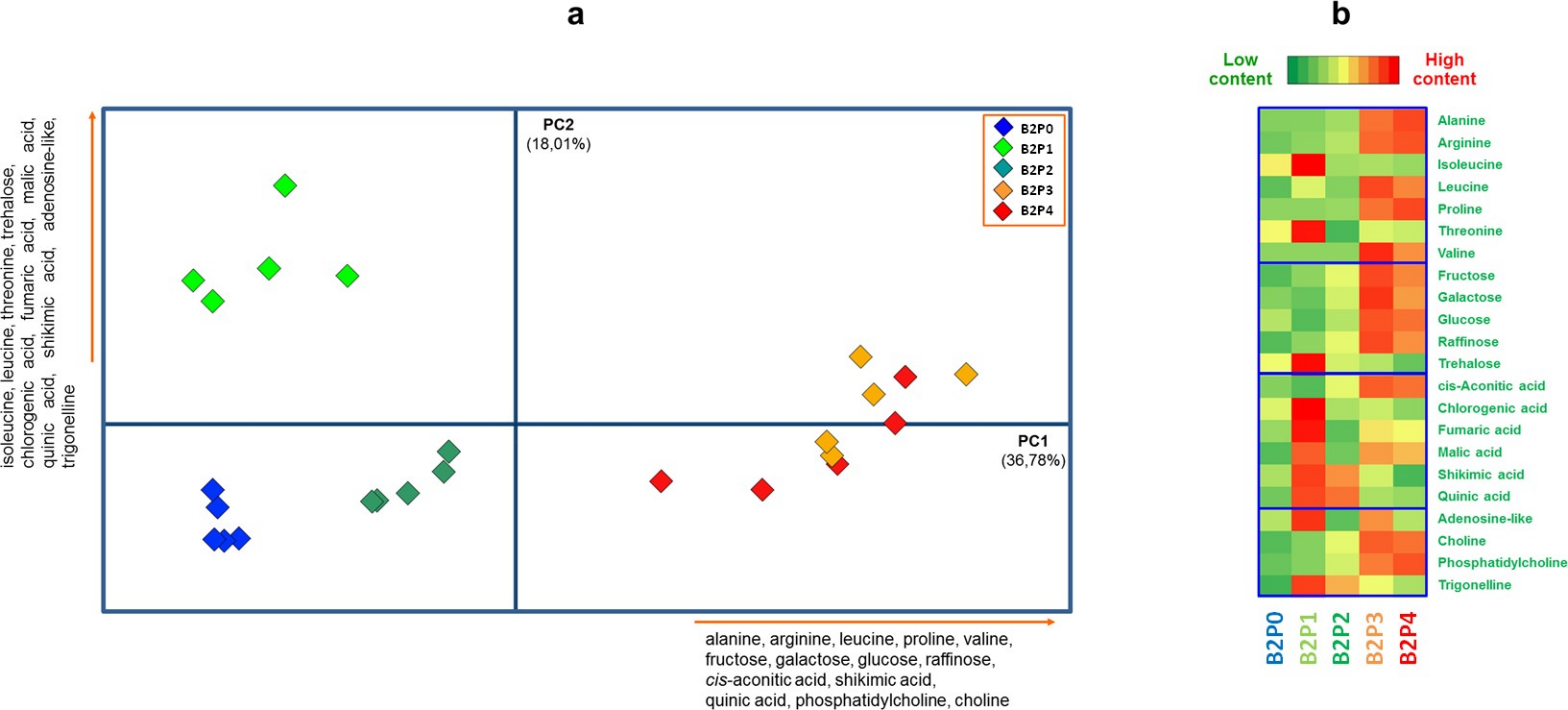
PCA and Heatmap of plant metabolites as a function of *Trichoderma* inoculum. (a) PCA score-plot based on GC-MS and ^1^H NMR data of leaves harvested from maize plants inoculated with *Trichoderma* (B2) and treated with different P-based fertilizers (P0-P4). Names and direction of most significant PCA loading vectors involved in the differentiation of treatments are reported along the score-plot borders. (b) Heatmap visualization of most discriminant variables.

The PCA of plants inoculated with *Trichoderma* in combination with different phosphate fertilizations explained 54.79% of the total variance (Fig 3A). In particular, PC1 (36.78% of the total variance) neatly differentiated plant samples treated with *Trichoderma* and the two composts (B2P3, B2P4), because of a greater amount of glucose, fructose, raffinose, cis-aconitic acid, choline, phosphatidylcholine, isocitric acid, alanine, arginine, proline and valine. In particular, the high concentration of glucose and fructose can be related to the photosynthesis process, and to an increased content of nutrients in plant leaves [43]. In fact, the greatest P and N increase observed in B2P3 and B2P4 plant leaves (Fig 1C and 1D) indicate that the metabolic findings are related to an improved plant nutritional status, that can be accounted to an efficient fungus-plant-compost synergism and a consequent increased of photosynthetic activity and shoots growth. It has been already pointed out [22] that the up-regulation of carbohydrate metabolism and photosynthesis is induced by the enhanced growth response due to the root colonization by *Trichoderma*.

Moreover, the detection of abundant levels of amino acids such as alanine, arginine and valine in the composts treatments suggests an enhanced synthesis of proteins that is common in a period of vegetative development, such as that occurred at sampling of these plants.

The combined effect of compost with *Trichoderma* determines a large concentration of phosphatidylcholine and of the intermediate choline, which well correlate with the increased absorption of P in plant. In fact, the content of the phosphatidylcholine, that is the main constituent of membrane lipids, changes according to the P bioavailability on plants [44].

The presence of metabolites like raffinose and proline in B2P3 and B2P4 plant samples may also suggest a stress signal. In particular, raffinose is assumed to be synthesized in response to a range of biotic or abiotic stresses [45], although its accumulation in plant cells appears to be also due to a carbon storage mechanism [46]. Proline is also commonly recognized as being produced in large concentration in response to a variety of abiotic stresses, while its catabolism can provide a supply of energy to drive plant growth once the stress is relieved [47]. In addition, the expression of stress related metabolites may also be due to the enhanced resistance conferred by the *Trichoderma* activity [22]. Therefore, the plant root colonization by *Trichoderma* may account for both an increased growth, that is mediated by enhanced photosynthetic and respiratory rates, and a priming of the systemic induced resistance, that upregulates the expression of stress related metabolites. These mechanisms seem to well fit our positive results obtained by treating maize plants with *Trichoderma* inoculum in combination with compost P-fertilizers.

Conversely, PC2 (18.01% of the total variance) neatly discriminated the plants treated with both TSP and *Trichoderma* (B2P1). In fact, these treatments were discriminated along the positive values of PC2 and were associated to large concentrations of trigonelline, fumaric acid, shikimic acid, chlorogenic acid, quinic acid, malic acid, trehalose, adenosine-like, threonine, and isoleucine. In particular, trigonelline is reported to serve as a cell cycle regulator, while it may also accumulate in response to salt, oxidative and UV stress conditions [48]. In fact, trigonelline may be a potent inducer of defensive metabolism in plants, including glutathione metabolism, and the accumulation of secondary defense compounds [48]. The induced synthesis of defense metabolites is accompanied by large contents of chlorogenic, shikimic, and quinic acids, which play important roles in the synthesis of secondary metabolites [41].

Moreover, the considerable content of threonine and isoleucine for the B2P1 treatment, in concomitance with the reduced values of P, N and chlorophyll in the related leaves extracts, may also suggest a state of plant stress. In fact, threonine, together with methionine, serves as substrate for the isoleucine synthesis, that in turn binds to leucine and valine to form BCAAs, which are synthesized at large concentrations in response to abiotic stresses [49].

Despite the similarity in P and N content in plants of B2P1 and B2P2 treatments (Fig 1), their placement along the PC2 revealed a lower amount of all variables in B2P2 than in B2P1 (Fig 3), thereby suggesting that B2P1 plants, also according the reduced shoot biomass (Fig 1A), had been subjected to stronger stress conditions. In fact, symptoms of stressed plants were noted at 8 weeks after sowing and were attributed to a sudden decrease of P bioavailability that no longer ensured the plant nutritional needs, even under *Trichoderma* inoculation.

Also in the case of the slow-release RP phosphate fertilizer, the *Trichoderma* inoculation did not produce positive synergistic effects towards plant growth and P uptake, as shown by the negative values of dry shoot biomass and P content, which were noted for B2P2 plants at sampling time. On the other hand, the small N and P content detected in B2P0 plants (Fig 1B and 1C), is well related with the scarce concentration of all metabolites, as shown by the negative placement of this treatment samples along both PC1 and PC2 components. This observation strengthens the hypothesis of competition for the poor P resources between the *Trichoderma* OmG-08 strain and plants, thus leading to a lesser photosyntetic activity that consequently affects plant growth [16, 30, 50].

### Effects of molecular characteristics of composts on plants

The noted differences in shoot dry weight and P content for plants treated with the two different composts in presence of *Trichoderma* inoculation (Fig 1), suggests that the effects had to be accounted to the different molecular composition of the two composts. These results are in line with what was reported by Cozzolino et al. [51], who showed that a different molecular characteristics of compost affected not only plants growth but also the rhizosphere microbial activity.

The identification compounds present in composts P3 and P4, which may have differentially influenced the *Trichoderma* activity, was achieved by thermochemolysis coupled with GC-MS [29].

S4 Table shows the individual molecular components of both composts and their respective concentrations expressed in μg g^-1^ of dry weight, while the main class of compounds released during thermochemolysis with a focus on the lignin components are summarized in S5 Table. P3 showed a large concentration of lignin derivatives, fatty acids and alcohols, while P4 revealed a greater content of hydroxy and dioic acids (S5 Table). The main differences in terms of lignin components were due to a larger concentration of guaiacyl groups (G) in P3 than in P4, followed by p-hydroxyphenyl (P) and syringyl (S) units. The guaiacyl unit is indicative of a strong contribution from gymnosperm softwood, while the p-hydroxyphenyl derivatives suggest a dominance of grass lignin [29, 51]. Conversely, P4 was characterized by a large content of syringyl units followed by similar amounts of guaiacyl and p-hydroxyphenyl groups (S4 and S5 Tables).

The concomitant presence of syringyl and guaiacyl derivatives in both composts suggests a contribution of hardwood of perennial angiosperm [29, 51]. The most representative guaiacyl molecule in P3 was LgG18, that accounted for 33.3% of the total, while in P4 the benzoic acid, 3,4-diOMe, ME (Lg G6) corresponded to 29.4%. Conversely, the trans 4-OMe cinnamic acid ME (Lg P18) was the most abundant p-hydroxyphenyl homologue in P3 and P4, reaching 56.6 and 29.2%, respectively. In the case of syringyl units, the most abundant derivatives were the 3,4,5-triOMe benzoic acid ME (Lg S6), corresponding to 24.2% in P3, and cis-1-OMe-1-(3,4,5-triOMephenyl)-1-propene (Lg S10), representing 48.5% in P4. Such different distribution in lignin components may have directly affected both plants responses and *Trichoderma* bioactivity. Savy et al. [52] showed the ability of water-soluble lignins to stimulate the emergence of maize seedlings and their early growth. This phenomenon was attributed to a possible hormone-like effects exerted on plants by p-hydroxybenzoic, vanillic, syringic, gallic, and protocatechuic acid present in lignin [53, 54].

The specific molecular content of P3 compost can thus explain its plant growth stimulation greater than for P4. In fact, lignin molecules may have differently affected the bioactivity of *Trichoderma*, and determine the changes in the growth and P content of plants of B2P2 and B2P4 treatments. Moreover, specific lignin derivatives in P3 are likely to stimulate *Trichoderma* to release cellulolytic enzymes, thus enabling the transformation of lignocellulose components and favoring the mineralization of organically bound nutrients, which become bioavailable in the rhizosphere [55, 56]. In fact, it has been ascertained that some *Trichoderma* species are known to produce cellulases and depend on degrading dead organic matter and wood. Furthermore, recent studies on *Trichoderma* genomes suggest that, although with different efficiency, the ability to degrade cellulose is present in the whole genus [18].

P3 also showed a great amount of fatty acids methyl esters (FAME) in which C16 and C18 chains were the most represented homologues (18.8 and 27.3%, respectively). Although to a lesser extent, C16 fatty acids were the predominant also in P4 (21.8%), while the second most abundant compound was C22 (S4 Table). In line with literature data [57], the anteiso C15 FAME and the cy C19 FAME, at different concentrations (S4 Table), were the most representative microbial fatty acids of P3 and P4. A further difference between the two composts was the greater presence of medium-long-chain alcohols in P3 than in P4. The different content and distributions of these compounds in the two composts may then justify the diverse *Trichoderma* bioactivity with a consequent repercussion on plants response.

## Conclusions

In this work we evaluated, in a pot soil experiment, the effect of *Trichoderma* inoculation on the growth and nutrient uptake of *Zea mays* plants treated either with or without different inorganic and organic P fertilizers. Besides the phenological appraisal of biomass and nutrient content, we investigated the changes caused by the fertilizer treatments on the polar metabolites extracted from maize plants and detected by both GC-MS and ^1^H NMR techniques.

All applied treatments with different P sources were very effective in influencing both the metabolic profiles and the P uptake of treated plants. However, the metabolic responses varied with the type of P fertilizer and whether or not the amendments were conducted in combination with the *Trichoderma* inoculum.

In particular, the combined use of *Trichoderma* OmG-08 and compost (B2P3 and B2P4), significantly improved the plant P uptake and the abundance of metabolites related to photosynthesis with positive repercussions on the produced shoots dry biomass, as compared to controls (B0P3, B0P4). However, the noted differences in shoot dry weight and P content for B2P3 samples in respect to B2P4 plants, suggest that there was also a different response of maize plants to the type of applied compost due to a varied molecular composition.

Conversely when combined with mineral fertilizers, the *Trichoderma* OmG-08 negatively affect the plants growth. Also the metabolic and the nutritional parameters observed in B2P1 and B2P2 treatments were worse than for B0P1 and B0P2. The B2P0 plants were found to be adversely influenced by the inoculation with *Trichoderma*, most probably because of the competition for the available rhizosphere nutrients between plants and microorganism.

Hence, our results confirm that the combination of *Trichoderma* OmG-08 with composts fertilizer may provide a valuable alternative to satisfy the plant P demand, thus reducing the use of the scarce and environmentally unsustainable mineral P fertilizers.

## Supporting information

S1 Table Main chemical-physical soil properties. (DOCX)

S2 Table List of primary metabolites from maize leaves identified by ^1^H NMR. (DOCX)

S3 Table List of primary metabolites from maize leaves identified by GC-MS. (DOCX)

S4 Table Thermochemolysis products released by bulk composts. (DOCX)

S5 Table Yields (μg g^-1^ dw) and classes of main thermochemolysis products released from compost samples (cow-manure P3, horse-manure P4). (DOCX)
