# Supplementary material for: An alternative to mineral phosphorus fertilizers: The combined effects of Trichoderma harzianum and compost on Zea mays, as revealed by 1H NMR and GC-MS metabolomics
Source: PLoS One. 2018 Dec 27;13(12):e0209664. doi: 10.1371/journal.pone.0209664 (PMC6307717; doi:10.1371/journal.pone.0209664)
Supplement: S1 Table — (DOCX) [file pone.0209664.s001.docx]

**S1 Table**

Main chemical-physical soil properties

| pH (CaCl_2_) |  | 7.3 |
| --- | --- | --- |
| Sand | % | 19.0 |
| Silt | % | 44.5 |
| Clay | % | 36.5 |
| Total C | % | 1.34 |
| Total N | % | 0.11 |
| P Olsen | mg kg^-1^ | 12 |
