# Supplementary material for: An alternative to mineral phosphorus fertilizers: The combined effects of Trichoderma harzianum and compost on Zea mays, as revealed by 1H NMR and GC-MS metabolomics
Source: PLoS One. 2018 Dec 27;13(12):e0209664. doi: 10.1371/journal.pone.0209664 (PMC6307717; doi:10.1371/journal.pone.0209664)
Supplement: S2 Table — (DOCX) [file pone.0209664.s002.docx]

**S2 Table**

List of primary metabolites from maize leaves identified by ^1^H NMR

| **Amino acids** | **Assignment** | **δ (^1^H)** | **Multiplicity** |
| --- | --- | --- | --- |
| Alanine | β−CH_3_ | 1.48 | doublet |
| Arginine | β−CH_2_ | 1.72 | multiplet |
| Arginine | β−CH_2_ | 1.65 | multiplet |
| γ−Aminobutyric acid (GABA) | γ−CH_2_ | 3.01 | triplet |
| γ−Aminobutyric acid (GABA) | α−CH_2_ | 2.28 | triplet |
| Glutammic acid | γ−CH_2_ | 2.34 | triplet |
| Histidine | C4H ring | 7.78 | singlet |
| Histidine | C2H ring | 7.05 | singlet |
| Isoleucine | γ'−CH | 1.45 | multiplet |
| Isoleucine | γ−CH | 1.25 | multiplet |
| Isoleucine | γ−CH_3_ | 1.02 | doublet |
| Isoleucine | δ−CH_3_ | 0.93 | triplet |
| Leucine | γ−CH | 1.69 | multiplet |
| Leucine | δ'−CH_3_ | 0.96 | doublet |
| Phenylalanine | CH3,5 | 7.44 | multiplet |
| Phenylalanine | CH2,6 | 7.31 | multiplet |
| Proline | β−CH_2_ | 2.32 | multiplet |
| Threonine | γ−CH**_3_** | 1.32 | doublet |
| Tryptophan | C4H ring | 7.72 | doublet |
| Tryptophan | C7H ring | 7.54 | doublet |
| Tryptophan | C6H ring | 7.28 | triplet |
| Tryptophan | C5H ring | 7.20 | triplet |
| Tyrosine | C2,6H ring | 7.18 | doublet |
| Valine | γ'−CH_3_ | 1.04 | doublet |
| Valine | γ'−CH_3_ | 1.04 | doublet |
| Valine | γ−CH_3_ | 0.99 | doublet |
| **Carbohydrates** | **Assignment** | **δ (^1^H)** | **Multiplicity** |
| Fructose |  | 4.08 | multiplet |
| Fructose |  | 3.99 | douplet of dubleduplet |
| Fructose |  | 3.89 | doubleduplet |
| Fructose |  | 3.85 | multiplet |
| Fructose |  | 3.80 | multiplet |
| Fructose |  | 3.79 | doubleduplet |
| Fructose |  | 3.70 | doubleduplet |
| Fructose |  | 3.65 | multiplet |
| Fructose |  | 3.57 | doublet |
| α−Galactose | C1H | 5.27 | doublet |
| β−Galactose | C1H | 4.60 | doublet |
| α-Glucose | C1H | 5.24 | doublet |
| β−Glucose | C1H | 4.64 | doublet |
| β−Glucose | C1H | 4.64 | doublet |
| Glucose |  | 3.90 | multiplet |
| **S2 Table** Continued |  |  |  |
| **Carbohydrates** | **Assignment** | **δ (^1^H)** | **Multiplicity** |
| Glucose |  | 3.84 | multiplet |
| Glucose |  | 3.83 | multiplet |
| Glucose |  | 3.77 | multiplet |
| Glucose |  | 3.72 | doubleduplet |
| Glucose |  | 3.54 | doubleduplet |
| Glucose |  | 3.51 | triplet |
| Glucose |  | 3.42 | triplet |
| Glucose |  | 3.40 | doubleduplet |
| Glucose |  | 3.24 | triplet |
| Glucose-6-phosphate |  | 5.58 | doublet |
| Inositol |  | 4.05 | triplet |
| Inositol |  | 3.61 | triplet |
| Inositol |  | 3.52 | doubleduplet |
| Raffinose | Gal1H | 5.00 | doublet |
| Raffinose | Fru3H | 4.23 | doublet |
| Sucrose | Glc1H | 5.42 | doublet |
| Sucrose | FruCH_2_-1' | 3.68 | singlet |
| Trehalose | C1H | 5.20 | doublet |
| **Nitrogenous compounds** | **Assignment** | **δ (^1^H)** | **Multiplicity** |
| Adenosine-like |  | 8.34 | singlet |
| Adenosine-like |  | 8.27 | singlet |
| Adenosine-like |  | 8.11 | doublet |
| Adenosine-like |  | 5.99 | doublet |
| UMP | C1'H ribose | 8.11 | doublet |
| UMP | C6 ring | 5.99 | doublet |
| Choline | N-CH_3_ | 3.21 | singlet |
| Dimethylamine | CH_3_ | 2.73 | singlet |
| Glicine betaine | N-CH_3_ | 3.28 | singlet |
| Phosphatidylcholine | CH_3_ | 3.27 | singlet |
| Trigonelline (HA) | HA | 9.13 | singlet |
| Trigonelline (HB, HC) | HB,HC | 8.86 | doublet |
| Trigonelline (HD) | HD | 8.06 | doublet |
| Trimethylamine | CH_3_ | 2.91 | singlet |
| **Organic acids** | **Assignment** | **δ (^1^H)** | **Multiplicity** |
| Chlorogenic acid |  | 7.6 | doublet |
| Chlorogenic acid |  | 7.13 | doublet |
| Chlorogenic acid |  | 6.87 | doublet |
| Chlorogenic acid |  | 6.35 | doublet |
| Chlorogenic acid |  | 5.31 | tripledublet |
| Cis-aconitic acid | CH=C- | 6.65 | singlet |
| Cis-aconitic acid | CH_2_ | 3.49 | singlet |
| Formic acid | HCOOH | 8.46 | singlet |
| Fumaric acid | α,β CH=CH | 6.53 | singlet |
| **S2 Table** Continued |  |  |  |
| **Organic acids** | **Assignment** | **δ (^1^H)** | **Multiplicity** |
| Isocitric acid |  | 2.96 | quartet |
| Isocitric acid |  | 2.46 | doublequartet |
| Maleic acid | α,β CH=CH | 6.00 | singlet |
| Malic acid | α−CH | 4.32 | doubledublet |
| Malic acid | β−CH | 2.68 | doubledublet |
| Malic acid | β−β'−CH_2_ | 2.38 | doubledublet |
| Quinic acid | H3 | 4.131 | quartet |
| Quinic acid | H5 | 4.008 | dublet of doubledublet |
| Quinic acid | H4 | 3.54 | doubledublet |
| Quinic acid | H6' | 2.04 | dublet of doubledublet |
| Quinic acid | H2 | 1.97 | doubledublet |
| Quinic acid | H2' | 1.94 | duplet of doubledublet |
| Quinic acid | H6 | 1.88 | doubledublet |
| Shikimic acid |  | 6.42 | multiplet |
| Shikimic acid |  | 4.38 | triplet |
| Shikimic acid |  | 2.77 | doubledublet |
| Shikimic acid |  | 2.73 | doubledublet |
| Shikimic acid |  | 2.18 | double of douplequartet |
| Succinic acid | α−β−CH_2_ | 2.41 | singlet |
