# Supplementary material for: An alternative to mineral phosphorus fertilizers: The combined effects of Trichoderma harzianum and compost on Zea mays, as revealed by 1H NMR and GC-MS metabolomics
Source: PLoS One. 2018 Dec 27;13(12):e0209664. doi: 10.1371/journal.pone.0209664 (PMC6307717; doi:10.1371/journal.pone.0209664)
Supplement: S3 Table — (DOCX) [file pone.0209664.s003.docx]

**S3 Table.**

List of primary metabolites from maize leaves identified by GC-MS.

| n° | **Metabolites ^a^** | **R.t. ^b^** | **MS fragmentation (m/z)** | **References ^c^** |
| --- | --- | --- | --- | --- |
| 1 | Lactic acid | 4.77 | 52-73-75-117-147-149 | NIST |
| 2 | Alanine | 5.28 | 73-75-116-117-147-190 | STD,MPI |
| 3 | Oxalic acid | 5.7 | 73-74-75-147-148-149 | NIST |
| 4 | Phosphoric acid | 7.07 | 73-133-211-225-299-300 | NIST |
| 5 | Glycerol tris TMS | 7.09 | 73-117-147-205-299 | NIST |
| 6 | Succinic acid | 7.51 | 73-147-172-247 | NIST |
| 7 | Glyceric acid | 7.65 | 73-103-147-189-292 | NIST |
| 8 | Itaconic acid | 7.78 | 73-147-215-259 | NIST |
| 9 | Serine | 7.94 | 73-75-116-147-204-218 | STD,MPI |
| 10 | Threonine | 8.18 | 73-75-130-147-218 | STD |
| 11 | Maleic acid | 8.32 | 73-75-133-147-148-149 | NIST |
| 12 | 4-Ketaglucose | 8.89 | 73-103-147-204 | NIST |
| 13 | Arabino-hexose-2-ulose | 8.98 | 73-103-147-205-234-262 | NIST,MPI |
| 14 | Methylmaleic acid | 9.07 | 73-147-189-233-265 | NIST,MPI |
| 15 | Aspartic acid | 9.35 | 73-75-100-147-232 | STD |
| 16 | succinic anhydride | 9.39 | 73-156-255 | NIST |
| 17 | 4-aminobutyric acid | 9.47 | 73-130-175-205-231-260 | NIST,STD |
| 18 | Tetronic acid | 9.65 | 73-147-220-292 | NIST |
| 19 | Glutaric acid | 9.81 | 73-147-198-304 | STD |
| 20 | Glutamine | 10.16 | 73-75-128-147-156-246 | NIST,MPI |
| 21 | Xylonic acid | 10.43 | 73-117-147-175-217-244 | NIST |
| 22 | Xylulose | 10.66 | 73-103-147-217-307 | NIST |
| 23 | Ribitol IS | 10.95 | 73-103-147-217-319 | STD |
| 24 | cis Aconitic acid | 11.14 | 73-147-229-285-375 | NIST,MPI |
| 25 | Cinnamic acid | 11.42 | 73-147-204-245 | STD,MPI |
| 26 | Shikimic acid | 11.55 | 73-147-204-255 | NIST,MPI |
| 27 | sugar | 11.58 | 73-147-205-217 | NIST |
| 28 | Isocitric acid | 11.63 | 73-147-273-363 | NIST |
| 29 | Quinic acid | 11.92 | 73-147-191-255-345 | NIST,MPI |
| 30 | Fructose | 12.00 | 73-129-133-147-217-218 | STD |
| 31 | Fructose | 12.06 | 73-129-133-147-217-219 | STD |
| 32 | Galactose | 12.14 | 73-147-205-217-229 | STD |
| 33 | Glucose | 12.18 | 73-129-147-149-157-217 | STD |
| 34 | Glucose | 12.32 | 73-129-147-149-157-218 | STD |
| 35 | UN | 12.54 | 73-147-219-292 | NIST |
| 36 | UN | 12.97 | 73-147-221-449 | NIST |
| 37 | Mucic acid | 13.15 | 73-147-217-292-333 | NIST |
| 38 | UN | 13.35 | 73-103-147-221-449 | NIST |
| 39 | Myo-Inositol | 13.47 | 73-147-191-217-221-305 | STD,MPI |
| 40 | Floridoside | 13.73 | 73-103-147-204-337 | NIST |
| 41 | Glucuronic acid | 13.86 | 73-147-205-217-292-375 | NIST |
| 42 | UN | 14.11 | 73-147-204-321-361 | NIST |

**S3 Table.** Continued

| n° | **Metabolites ^a^** | **R.t. ^b^** | **MS fragmentation (m/z)** | **References ^c^** |
| --- | --- | --- | --- | --- |
| 43 | sugar | 14.81 | 73-129-133-147-217-219 | NIST |
| 44 | UN | 15.12 | 73-147-204-321-361-406 | NIST |
| 45 | oligosaccaride | 15.52 | 73-147-217-361-450 | NIST |
| 46 | oligosaccaride | 15.53 | 73-147-217-361-450 | NIST |
| 47 | oligosaccaride | 16.25 | 73-147-217-361-450 | NIST |
| 48 | Sucrose | 16.47 | 73-169-217-243-361-362 | NIST |
| 49 | UN | 16.55 | 73-147-204-217-361 | NIST |
| 50 | D-Turanose | 17.35 | 73-147-217-361-450 | NIST |
| 51 | Maltose | 18.47 | 73-147-204-217-305-361 | NIST |
| 52 | UN | 19.05 | 73-147-219-255-345 | NIST |
| 53 | Melibiose | 19.45 | 73-103-129-204-217-361 | NIST |
| 54 | UN | 20.19 | 73-147-191-249-345-447 | NIST |
| 55 | Chlorogenic acid | 20.08 | 73-147-191-255-307-345 | STD,MPI |
| 56 | Raffinose | 22.35 | 73-147-129-205-217-361-437 | NIST |

**a)** UN= unknown

**b)** R.t.= Retention time (min);

**c)** NIST= mass spectra library NIST 05 (http://www.nist.gov); STD=standards; MPI=Max-Planck-Institute (Germany, http://csbdb.mpimp golm.mpg.de/csbdb/dbma/msri.html).
