# Supplementary material for: An alternative to mineral phosphorus fertilizers: The combined effects of Trichoderma harzianum and compost on Zea mays, as revealed by 1H NMR and GC-MS metabolomics
Source: PLoS One. 2018 Dec 27;13(12):e0209664. doi: 10.1371/journal.pone.0209664 (PMC6307717; doi:10.1371/journal.pone.0209664)
Supplement: S4 Table — (DOCX) [file pone.0209664.s004.docx]

**S4 Table**

Thermochemolysis products released by bulk composts.

| **R.t.^a^** | **Assignment^b^** | **Symbol^b^** | **P3^c^** | **P4^c^** |
| --- | --- | --- | --- | --- |
| 7.85 | 4-Methoxy, 1-vinylbenzene | Lg P3 | 256.6 | 218.6 |
| 8.04 | 1,2-Dimethoxy benzene | Lg G1 | 23.9 | 41.3 |
| 9.73 | 3,4-OMe toluene | Lg G2 | 90.8 | 117.0 |
| 11.37 | 1,2,3-tri-OMe benzene | Lg S1 | 35.1 | 58.3 |
| 11.78 | 4-OMe Acetophenone | Lg P5 | 89.3 | 28.5 |
| 11.86 | C10 FAME | Lip | 113.2 | tr |
| 12.28 | Benzoic acid, 4-methoxy, ME | Lg P6 | 71.0 | 35.5 |
| 12.73 | 3,4,5-triOMe benzene, 1-methyl | Lg S2 | 105.8 | 117.5 |
| 13.06 | Benzene, 4-ethenyl-1,2-diOMe | Lg G3 | 181.5 | 90.7 |
| 13.20 | 1,2,5-triOMe benzene | Lg S | 75.3 | 67.0 |
| 13.34 | Benzoic acid, 4-methoxy, ME | Lg P7 | 112.7 | 402.1 |
| 13.60 | Charboidrate derivative | Carb | tr | tr |
| 13.96 | 3,4,5-triOMe benzene, ME | Lg S2 | 151.8 | 128.3 |
| 14.24 | 1,2,5-triOMe benzene | Lg | 97.1 | tr |
| 14.95 | Benzoic acid, 4-methoxy, ME | Lg S2 | 79.9 | 23.6 |
| 15.17 | cis 1-(4-OMe phenyl)-1-OMe prop-1-ene | Lg P10 | 63.9 | 33.7 |
| 16.28 | 3,4-diOMe benzaldehyde | Lg G4 | 163.0 | 178.2 |
| 16.72 | trans 1-(4-OMe phenyl)-1-OMe prop-1-ene | Lg P11 | 102.8 | 253.9 |
| 17.06 | 3,4,5-tri-OMe styrene | Lg S3 | 58.0 | 69.3 |
| 17.39 | C12 FAME | Lip | 190.2 | 88.0 |
| 17.50 | cis 1-(3,4-diOMe phenyl)-2-OMe-ethene | Lg P12 | 49.8 | 28.6 |
| 18.02 | C9 dioic acid DIME | Mic | 614.1 | 600.3 |
| 18.54 | 3,4-diOMe acetophenone | Lg G5 | 157.2 | 92.5 |
| 19.32 | Benzoic acid, 3,4-diOMe, ME | Lg G6 | 335.9 | 441.7 |
| 19.51 | 3,4,5-tri-OMe benzaldehyde | Lg S4 | 118.0 | 101.8 |
| 20.18 | cis 1-(3,4-diOMe phenyl)-2-OMe-ethene | Lg G7 | 152.1 | 142.6 |
| 20.54 | trans 1-(3,4-diOMephenyl)-2-OMe-ethene | Lg G8 | 131.6 | 66.7 |
| 21.55 | trans 4-OMe cinnamic acid, ME | P18 | 1013.1 | 412.4 |
| 22.69 | 3,4,5-triOMe benzoic acid ME | Lg S6 | 354.6 | 352.7 |
| 22.87 | C14 FAME | Lip | 215.4 | 102.9 |
| 23.22 | 1-(3,4-diOMephenyl)-3-OMe prop-1-ene | Lg G13 | 96.6 | 86.3 |
| 24.09 | cis-1-(3,4,5-triOMeyphenyl)-2-OMe ethylene | Lg S7 | 164.9 | 115.7 |
| 24.51 | C15 iso FAME | Mic | 387.8 | 178.8 |
| 24.63 | C15 anteiso FAME | Mic | 562.7 | 426.6 |

**S4 Table** Continue

| **R.t.^a^** | **Assignment^b^** | **Symbol^b^** | **P3^c^** | **P4^c^** | |
| --- | --- | --- | --- | --- | --- |
| 24.86 | cis-1-OMe-1-(3,4,5-triOMephenyl)-1-propene | Lg S10 | 305.0 | 950.5 |  |
| 25.48 | C15 n FAME | Lip | 189.8 | tr |  |
| 26.88 | 3-(3,4-diOMe phenyl)-3-propenoic acid, ME | Lg G18 | 690.9 | 175.9 |  |
| 26.94 | trans-1,3-diOMe-1-(3,4-diOMe phenyl)-1-propene | Lg G19 | tr | 39.3 |  |
| 27.42 | C16:1 FAME | Lip | 236.4 | tr |  |
| 27.99 | C16 FAME | Lip | 1740.4 | 482.5 |  |
| 29.46 | C17 iso FAME | Mic | 119.5 | 54.1 |  |
| 29.65 | C17 anteiso FAME | Mic | 133.5 | 36.4 |  |
| 30.36 | C17 n FAME | Mic | 156.7 | tr |  |
| 32.01 | C18:1 FAME | Lip | 619.2 | 181.1 |  |
| 32.2 | C18:1 FAME | Lip | 349.3 | tr |  |
| 32.65 | C18 FAME | Lip | 2532.7 | 191.7 |  |
| 33.27 | C16, 16 OMe, FAME | Lip | tr | 323.6 |  |
| 34.56 | cy C19 FAME | Mic | 211.7 | 624.1 |  |
| 35.38 | C16 dioic acid DIME | Lip | tr | 1020.6 |  |
| 36.11 | C16, 10-16 di OMe, FAME | Lip | tr | 1317.5 |  |
| 36.91 | C18:1, 18OMe, FAME | Lip | 270.5 | 482.5 |  |
| 37.01 | C20 FAME | Lip | 183.6 | 57.2 |  |
| 38.23 | Dioic acid | Lip | 1588.3 | 1450 |  |
| 38.87 | C18:1, dioic acid, DIME | Mic | 524.3 | 920.9 |  |
| 40.43 | alkane | Lip | tr | tr |  |
| 40.99 | C22 FAME | Lip | 461.0 | 311.0 |  |
| 41.51 | C20, 20-OMe, FAME | Lip | 24.0 | 59.5 |  |
| 41.79 | C18, 9,10,19 triOMe, FAME | Lip | 398.3 | 711.0 |  |
| 42.33 | alkane | Lip | tr | tr |  |
| 42.87 | C23 FAME | Lip | 136.5 | 104.2 |  |
| 43.03 | C18, 9,10,19 triOMe, FAME | Lip | 535.8 | 695.8 |  |
| 44.71 | C24 FAME | Lip | 449.3 | 215.6 |  |
| 45.22 | C22, 22-OMe, FAME | Lip | 111.8 | 323.8 |  |
| 46.45 | C26-OMe | Lip | 434.6 | 77.2 |  |
| 46.93 | C24, 2-OMe, FAME | Mic | 142.8 | 88.6 |  |
| 48.18 | C26 FAME | Lip | 875.6 | 256.3 |  |
| 48.66 | C25, 2-OMe, FAME | Mic | 225.2 | 155.1 |  |
| 49.81 | C28-OMe | Lip | 322.1 | 55.6 |  |

**S4 Table** Continue

| **R.t.^a^** | **Assignment^b^** | **Symbol^b^** | **P3^c^** | **P4^c^** |
| --- | --- | --- | --- | --- |
| 50.21 | C30-OMe | Lip | 251.1 | tr |
| 52.85 | C28-FAME | Lip | 518.3 | 160.1 |
| 54.53 | C30-OMe | Lip | 288.6 | 83.8 |
| 56.66 | C30-FAME | Lip | 292.6 | 59.6 |

**a)** R.t.= Retention time (min);

**b)** Carb. = Carbohydrates; FAME = fatty acid methyl ester; Lg.= Lignin; Lip.= lipid; Me = methyl ester; OMe = Methoxy; Mic.= Microbial.

P = p-hydroxyphenyl; G = guaiacyl (3-methoxy, 4-hydroxyphenyl); S = syringyl (3,5-dimethoxy, 4-hydroxyphenyl).

**c)** Composts from cow (P3) and horse manure (P4). Results expressed as μg g^-1^ of dry weight, tr = traces.
