# Supplementary material for: An alternative to mineral phosphorus fertilizers: The combined effects of Trichoderma harzianum and compost on Zea mays, as revealed by 1H NMR and GC-MS metabolomics
Source: PLoS One. 2018 Dec 27;13(12):e0209664. doi: 10.1371/journal.pone.0209664 (PMC6307717; doi:10.1371/journal.pone.0209664)
Supplement: S5 Table — (DOCX) [file pone.0209664.s005.docx]

**S5 Table**

Yields (μg g^-1^ dw) and classes of main thermochemolysis products released from compost samples (cow-manure P3, horse-manure P4).

| **Compounds** | **P3** | **P4** |
| --- | --- | --- |
| Lignin | 5329 | 4870 |
| *Guaiacil structure (G)* | *2073.4* | *1500.8* |
| *Syringyl structure (S)* | *1466.1* | *1961.1* |
| *p- Hydroxyphenyl structure (P)* | *1789.4* | *1408.4* |
| FAME C10–C30 | 9260 | 2210 |
| *Medium-chain* (C10-C15) | *708.6* | *191.1* |
| *Long-chain* (C16-C30) | *855.2* | *2019.4* |
| Microbial fatty acids C15–C24 (C17) | 1783 | 1564 |
| Hydroxy acids C9–C24 (C18:1) | 1340 | 3913 |
| Dioic acids | 2727 | 4056 |
| Alcohols (C26–C30) | 1296 | 217 |
| AD/AL_G_ | 2.06 | 2.47 |
| AD/AL_S_ | 3.00 | 3.46 |

Total range varying from Ci to Cj; compounds in parentheses are the most dominant homologues. *FAME* fatty acid methyl ester, *AD/AL_G_ =* G6/G4, *AD/AL_S_* =S6/S4, employing the aldehydic (G4, S4) and acidic forms (G6, S6) of guaiacyl (G) and syringyl (S) structures.
